# Effect of Intravenous Injection of Magnesium Sulphate on Intraoperative End-Tidal CO_2_ Level and Postoperative Pain in Laparoscopic Cholecystectomy

**DOI:** 10.5812/aapm-135189

**Published:** 2023-12-31

**Authors:** Mahboobeh Akhondi, Ali Sarkoohi

**Affiliations:** 1 Department of Anesthesiology, Rafsanjan University of Medical Sciences, Rafsanjan, Iran; 2 Ali Ibn Abitaleb Educational and Tretment Hospital, School of Medicine, Rafsanjan University of Medical Sciences, Rafsanjan, Iran

**Keywords:** Laparoscopy, Cholecystectomy, Postoperative Pain, Magnesium Sulphate

## Abstract

**Background:**

Pain control and stabilizing hemodynamic indices are serious medical challenges, especially in anesthesia. Laparoscopic surgery is increasing in the world, and cholecystectomy surgery is no exception.

**Objectives:**

This study investigated the effect of intravenous (IV) magnesium sulfate injection on intraoperative end-tidal CO_2_ (ETCO_2_) levels and postoperative pain in laparoscopic cholecystectomy.

**Methods:**

This is a clinical trial. The sample size was calculated to be 64 people who were selected among the patients who were candidates for laparoscopic surgery by convenience sampling. They were randomly assigned to intervention and control groups. The intervention group received magnesium sulfate (50 mg/kg) and normal saline (100 mL) within 1 h. The control group only received normal saline (100 mL). Systolic and diastolic blood pressures, ETCO_2_ level, heart rate, arterial oxygen saturation, pain level, and narcotic analgesics in recovery were measured 2, 6, 12, and 24 h after surgery. The data were analyzed using 1-way analysis of variance (ANOVA) and repeated measures analysis.

**Results:**

The mean of systolic blood pressure and ETCO_2_ during recovery in the intervention group were less than the control group (P = 0.029 and P = 0.015). In the intervention group, analgesic consumption in recovery and 6 h after surgery was less than the control group (P < 0.001). The mean pain score in the intervention group in recovery and 2, 6 (P < 0.001), and 12 h (P = 0.038) after surgery was significantly lower than the control group.

**Conclusions:**

Magnesium sulfate can be a suitable and safe supplement to reduce pain after surgery and reduce the use of narcotics. The current conclusion should be investigated on a larger scale of patients, with extended monitoring for postoperative pain over a longer period of time.

## 1. Background

Laparoscopic cholecystectomy is a minimally invasive surgical procedure used to remove a patient's gallbladder. Since the early 1990s, this method has largely replaced open cholecystectomy. Laparoscopic cholecystectomy is currently used for the treatment of acute or chronic cholecystitis, symptomatic kidney stones, biliary dyskinesia, acallous cholecystitis, gallstone pancreatitis, and gallbladder masses or polyps.

Postoperative pain is a complex physiological reaction to tissue damage. The main concern of patients undergoing surgeries is the postoperative pain that they would experience. Postoperative pain causes acute adverse physiological impacts associated with manifestations on multiple organ systems, possibly causing significant morbidity. The pain that limits walking after surgery and increases stress-induced coagulation may increase the risk of deep vein thrombosis (DVT). Catecholamines released in response to pain may cause tachycardia and systemic hypertension, causing myocardial ischemia in predisposed patients. Surgery and postoperative planning aim to reduce the pain level. Proper control of postoperative pain improves postoperative rehabilitation, short- and long-term recovery, and postoperative quality of life (1). Narcotic analgesics are associated with various complications (such as respiratory depression), causing insufficient dose prescription of narcotics that do not well control the pain. Finding more effective drugs reduces the pain and costs for patients and hospitals while increasing postoperative satisfaction and quality of life (2, 3).

Despite all the advantages of laparoscopic surgeries, postoperative pain remains a basic concern for patients undergoing such surgeries. This medical problem may cause clinical and mental changes and increased complications, mortality rate, and costs, reducing the quality of life (4). Inefficient postoperative pain management may cause DVT, pulmonary embolism, coronary artery stress, atelectasis, pneumonia, poor wound healing, insomnia, and demoralization (5, 6). Carbon dioxide gas is usually blown into the abdomen to create pneumoperitoneum in laparoscopy (7, 8), moving the abdomen contents away from the intended site and providing a better background and visibility for the surgeon for required procedures. However, system absorption of CO_2_ in the peritoneal cavity causes hypercarbia (9). On the other hand, when pneumoperitoneum is associated with a patient’s Trendelenburg with an angle of 15 - 20°, it significantly impacts the patient’s hemodynamics (10) by suddenly increasing the arterial blood pressure, increasing the peripheral vascular resistance, and reducing the cardiac output (11). Notably, a sudden increase in arterial blood pressure and heart rate may cause multiple damages to the patient, which can be irreparable in patients with underlying heart disease (12). In the meantime, magnesium sulfate can be a suitable approach to reducing cardiac risks due to preventing catecholamine release in the adrenal gland and peripheral nerve endings (13). The challenge faced by anesthesiologists in laparoscopic surgeries is the effect of CO_2_ gas on patients during pneumoperitoneum. Magnesium sulfate is increasingly used due to its effect on hemodynamic stability. Generally, the magnesium effect is related to the interference in membrane ca-ATPase and Na-K ATPase activation, playing a key role in the membrane exchange of ions. Consequently, it can be argued that magnesium sulfate is considered a cell membrane modifier. Moreover, the inhibitory effect of magnesium on calcium causes vasodilation and prevents vasospasm. On the other hand, magnesium reduces catecholamine release by sympathetic stimulation, reducing response to postoperative stress (13, 14). Magnesium sulfate is an NMDA antagonist receptor (*N*-methyl-D-aspartate) and calcium channel blocker. NMDA receptors play a vital role in pain transmission in the central and peripheral nervous systems, causing acute pain in the body. By blocking calcium channels, they prevent the transmission of pain nerve impulses (15).

A meta-analysis supports the idea that magnesium sulfate can be prescribed to provide stable anesthesia without prescribing opioids. Since this study was conducted on gynecologic surgeries, and the positive effect of magnesium sulfate on the provision of stable anesthesia has been confirmed, this drug can also be safely used in all laparoscopic surgeries, such as gynecologic operations on Trendelenburg patients. This meta-analysis study confirms the results of this study on the positive effect of magnesium sulfate injection (16). A study showed that prescribing magnesium increases the effect of local anesthetics (17). Based on electron microscopy, the researchers found that intrathecal administration of magnesium sulfate causes neurodegeneration (18). Magnesium acts as an antagonist for NMDA receptors and can relieve pain. The pain relief effect of magnesium has been confirmed in intra- and postoperative periods (19-21).

## 2. Objectives

Since recent studies have emphasized the positive pretreatment effects of magnesium sulfate in managing and controlling surgery-induced pain, this study investigated the effect of intravenous (IV) injection of magnesium sulfate on the intraoperative end-tidal CO_2_ (ETCO_2_) level and postoperative pain in laparoscopic cholecystectomy.

## 3. Methods

This double-blind, randomized clinical trial was approved by the Ethics Committee of Rafsanjan University of Medical Sciences (IR.RUMS.REC.1399.235) and the Iranian Registry of Clinical Trials (IRCT20210302050549N1). The participants included all patients who were candidates for laparoscopic cholecystectomy who visited Ali Ibn Abitaleb Hospital in Rafsanjan city in 2021. A sample volume of 64 was calculated using the convenience sampling method and enrolled in the study.

Inclusion criteria were informed consent to participate in the study, class I and II anesthesia, and an age range of 20 - 60 years. Non-inclusion criteria were a history of drug abuse, neuromuscular diseases, liver and kidney failure, heart disease, cholecyst surgery, drug sensitivity to magnesium sulfate, chronic obesity, and an ejection fraction larger than 40%. Exclusion criteria were over 20% reduction in the blood pressure or heart rate during anesthesia. The visual analog scale (VAS) was used to measure the pain level. The visual analog scale is a numerical observational scale to express the pain level in patients ranging from 0 to 10 (0 indicates the lack of pain, and 10 indicates unbearable pain) (22). After describing the study goals and obtaining informed consent, the hospitalized patients were trained on how to use this scale.

The body mass index (BMI) was calculated by measuring the height and weight after entering the operating room. Systolic and diastolic blood pressures were measured by an arm mercury sphygmomanometer cuff (AIPk-II) before and after surgery. The heart rate and the atrial oxygen saturation were measured before and after surgery, respectively, by cardiac monitoring (SAADAT, Alborz-25, Iran) and pulse oximetry monitoring. The ETCO_2_ level was measured and recorded by an anesthesiology resident using a capnograph. The general anesthesia technique was the same in all operations, and the patients received no prophylaxis. All surgeries were performed by a surgeon and an anesthesiologist. Anesthesia indication started with fentanyl (2.5 µg/kg) and nesdonal (5 mg/kg). Atracurium was used for intubation (0.4 - 0.5 mg/kg IVP over 60 s, then 0.08 - 0.1 mg/kg 20 - 45 min after the initial dose to maintain neuromuscular block). O_2_/N_2_O (50/50) and propofol infusion (100 µg/kg/min) were used to maintain anesthesia (23, 24).

People were placed in the intervention or control group by lottery, so it was determined that the first patient would be placed in the intervention group and the second patient in the control group, and this process continued until the last person. The intervention group received 50 mg/kg of magnesium sulfate diluted in 100 mL of 0.9% normal saline after endotracheal intubation for 15 min, but the control group received 100 mL of 0.9% normal saline immediately after tracheal intubation (25).

The bleeding rate and the volume of received liquids were recorded during surgery. The postoperative pain intensity was measured and recorded using VAS after recovery and 2, 6, 12, and 24 h after surgery. Systolic and diastolic blood pressures, heart rate, arterial oxygen saturation, and intraoperative ETCO_2_ level were recorded within 5-min intervals. The opioid amount consumed (in the case of pain score higher than 5) was recorded in the checklist after recovery and 6 and 12 h after anesthesia. The data were analyzed using SPSS version 21, as well as using 2-way analysis of variance (ANOVA) with repeated measures, Tukey's multiple comparisons, chi-square, and independent *t*-tests. A significance level of 5% was considered. It should be noted that our colleague who measured the hemodynamic indices and the data analysts were unaware of the grouping of subjects.

## 4. Results

The intervention group included 12 males (37.5%) and 20 females (62.5%), while the control group consisted of 6 males (18.8%) and 26 females (81.3%). There was no significant difference between the groups regarding gender (P = 0.095). There was also no significant difference between the 2 groups regarding mean age (P = 0.651) and BMI (P = 0.994; Table 1). In recovery, the mean ETCO_2_ level was significantly lower in the intervention group (30.2 ± 81.58) than in the control group (32.50 ± 2.98). In recovery, the systolic blood pressure of patients was also significantly lower in the intervention group (119.66 ± 15.75) than in the control group (128.12 ± 14.59). There was no significant difference between the 2 groups in recovery regarding diastolic blood pressure, heart rate, arterial oxygen saturation, the volume of received liquids, and surgery duration (P > 0.05; Table 2). 

**Table 1.** Demographic Indices of Patients Who Underwent Laparoscopic Cholecystectomy in the Intervention and Control Groups

Variables	Intervention Group	Control Group	P-Value
**Gender**			0.095 ^a^
Male	12 (37.5)	6 (18.8)	
Female	20 (62.5)	26 (81.3)	
**Age (y)**	45.44 ± 10.60	44.25 ± 10.28	0.651 ^b^
**BMI**	27.26 ± 3.01	27.25 ± 3.00	0.994 ^b^

Abbreviation: BMI, body mass index.

^a^ Chi-square test.

^b^ Independent *t*-test, P < 0.05.

**Table 2.** Hemodynamic Indices of Patients Who Underwent Laparoscopic Cholecystectomy in the Intervention and Control Groups

Variables	Intervention Group	Control Group	P-Value
**ETCO**_**2**_ ** pressure**	30.81 ± 2.58	32.50 ± 2.98	0.015 ^a^
**Systolic blood pressure (mmHg)**	119.66 ± 15.75	128.12 ± 14.59	0.029 ^a^
**Diastolic blood pressure (mmHg)**	76.31 ± 10.67	79.47 ± 9.62	0.219 ^a^
**Heart rate per minute**	80.11 ± 63.26	81.22 ± 9.52	0.821 ^a^
**O**_**2**_ **SAT**	99.0 ± 25.80	99.41 ± 0.71	0.413 ^a^
**Volume of received liquids (mL)**	1515.63 ± 200.17	1453.13 ± 232.77	0.254 ^a^
**Surgery duration (min)**	43.28 ± 9.55	46.41 ± 9.35	0.191 ^a^

Abbreviation: ETCO_2_, end-tidal CO_2_.

^a^ Independent *t*-test, P < 0.05.

In recovery, 40.6% in the intervention group and 93.7% in the control group received narcotic sedatives. Six hours after surgery, 31.2% in the intervention group and 90.6% in the control group received narcotic sedatives. Finally, 24 h after surgery, 87.5% in the intervention group and 81.3% in the control group received narcotic analgesics (Table 3). 

**Table 3.** The Frequency Distribution of Pethidine Injection in Patients Who Underwent Laparoscopic Cholecystectomy in the Intervention and Control Groups

Pethidine Injection Time	Intervention (n = 32)	Control (n = 32)
**In recovery**		
Yes	13 (40.6)	30 (93.7)
No	19 (59.4)	2 (6.3)
**6 h after surgery**		
Yes	10 (31.2)	29 (90.6)
No	22 (68.8)	3 (9.4)
**24 h after surgery**		
Yes	28 (87.5)	26 (81.3)
No	4 (12.5)	6 (18.8)

The repeated measures analysis was used to evaluate pain level variations in both groups in recovery up to 24 h after surgery. Given the significant impact of time, the pain level decreased in both groups with time from recovery to 24 h after surgery (P < 0.001). The significant impact of intervention indicates a significant difference between the 2 groups regarding the pain level, so the pain level was lower in the intervention group than in the control group (P < 0.001). The significant interaction of time and intervention indicates a significant difference in the pain level variations in both groups (P = 0.009; Table 4). 

**Table 4.** The Results of Repeated Measurements for Pain Level Variations in the 2 Groups of Patients Who Underwent Laparoscopic Cholecystectomy

Source of Variations	Sum of Squares	Degree of Freedom	Mean Square	F-Value	P-Value
**Time**	26.331	4	6.583	7.928	< 0.001
**Intervention × time**	13.644	2.995	4.556	3.952	0.009
**Intervention**	78.013	1	78.013	44.978	< 0.001
**Error**	107.538	62	1.734		

Given the significant impact of the intervention and the 2 studied groups, Tukey’s post-hoc test was used for paired comparison of the 2 groups. On average, the mean pain in the intervention group was 0.988 higher than the control group, which was statistically significant (P < 0.0001; Table 5). 

**Table 5.** The Results of the Post-Hoc Test

Groups		Mean Difference (I - J)	SE	P-Value
Intervention (group I)	Control (group J)	-0.988	0.147	< 0.0001

**Figure 1.**
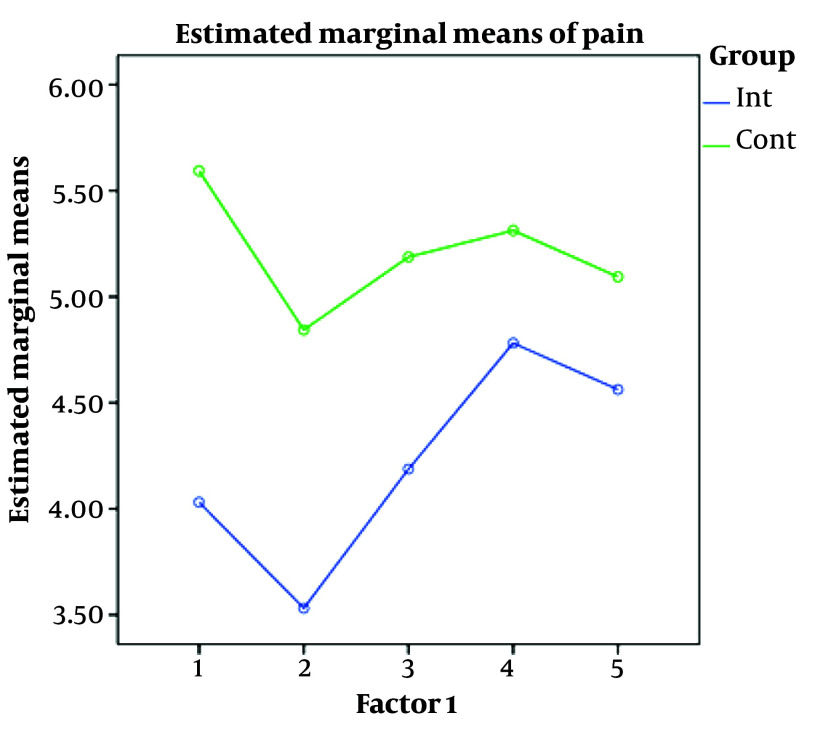
Pain level variations in both groups vs time

At all times, the mean pain level was less in the intervention group than in the control group. The mean pain level in both groups decreased over time. However, the pain level variations were almost identical in both groups and appeared as 2 parallel lines (Figure 1). 

## 5. Discussion

This study investigated the effect of IV magnesium sulfate injection on the intraoperative ETCO_2_ level and postoperative pain in laparoscopic cholecystectomy. The results showed a significant difference between the 2 groups regarding postoperative pain. However, this difference was not significant 24 h after surgery. The time variations of pain were significant in the intervention group but were insignificant in the control group. Moreover, drug consumption in recovery and 6 h after surgery was lower in the intervention group than in the control group. A meta-analysis examined intraoperative prescription of magnesium and postoperative pain in 25 clinical trials. The results showed a 24.4% reduction in morphine consumption and a reduction in the pain score 24 h after surgery (26). Su et al. showed that the intraoperative use of magnesium sulfate reduced the need for anesthetics (27). Dar et al. found that prescribing 50 mg/kg of magnesium sulfate reduced hemodynamic changes in laparoscopic surgeries (28). Consistent with our results, Mentes et al. reported reduced pain scores and narcotic dose in patients who underwent laparoscopic cholecystectomy in the magnesium sulfate group 0, 4, and 12 h after surgery (29). Radwan et al. concluded that the use of magnesium sulfate is rational and effective in reducing pain, is more physiological, and shortens convalescence after outpatient arthroscopic meniscectomy (30). Asadollah et al. confirmed the effectiveness of magnesium sulfate in reducing postoperative pain control following lower abdominal laparotomy (31). Consistent with our results, Kaur et al. found a positive effect of magnesium sulfate on reduced pain and consumption of analgesics after upper-extremity orthopedic surgery (32).

Magnesium sulfate improves the effect of local anesthesia on peripheral nerves and thus is used as a muscle relaxant for pain relief (33). Magnesium can have a pain-prevention effect before the onset of surgery-induced stimulation. Preventive analgesia that occurs by preventing the formation of the central sensitization process or what happens by cutting, inflammation, or both can be useful for patients. Many studies have shown the need for peri-anaoperative analgesics by magnesium prescription (34, 35). Our results are also consistent with those reported in the literature. Levaux et al. used 50 mg/kg of IV magnesium sulfate to reduce pain after major orthopedic spine surgery (36). Ghaffaripour et al. showed that the infusion of magnesium sulfate during laminectomy had no effect on patients' pain and opioid requirement during the first 24 h after surgery (37). The significant difference in postoperative pain disappeared after 24 h. Magnesium sulfate usually has a reduced effect because it usually takes effect immediately and can remain in the body's system for at least several hours and up to about 24 h. Consistent with our results, Bhatia et al. studied the effects of magnesium infusion on analgesia during cholecystectomy and reported no significant decrease in the amount of consumed morphine (38). One potential explanation for this controversy between our findings and previous studies is a difference in the patient population. Pain perception can be influenced by various factors, such as gender, psychological, personality, genetics, and ethnicity. Some ethnicities can tolerate pain better than others. In some cultures, enduring pain is considered a pleasant or acceptable experience (39, 40). In this study, the opioid consumed 24 h after surgery was lower in the magnesium sulphate group than in the control group. Based on the VAS values, the pain intensity scores in the group that received magnesium sulfate were significantly lower than those in the control group. Since abdominal surgeries are associated with a lot of pain, and this pain can lead to immobility and complications (such as constipation, infection, clot formation, and other complications), magnesium sulfate can be safely used to reduce pain after surgery to reduce the consumption of narcotics and prevent their side effects. The current conclusion needs to be investigated over a wider scale of patients, with extended monitoring for postoperative pain over a longer time frame.

### 5.1. Conclusions

Magnesium sulfate is a suitable and safe supplement to reduce pain after surgery and reduce the use of narcotics. The current conclusion should be investigated on a larger scale of patients, with extended monitoring for postoperative pain over a longer period of time.

## Clinical Trial Registration Code:

IRCT20210302050549N1.
